# Heritability Estimates of Body Size in Fetal Life and Early Childhood

**DOI:** 10.1371/journal.pone.0039901

**Published:** 2012-07-25

**Authors:** Dennis O. Mook-Kanamori, Catharina E. M. van Beijsterveldt, Eric A. P. Steegers, Yurii S. Aulchenko, Hein Raat, Albert Hofman, Paul H. Eilers, Dorret I. Boomsma, Vincent W. V. Jaddoe

**Affiliations:** 1 The Generation R Study Group, Erasmus Medical Center, Rotterdam, The Netherlands; 2 Department of Epidemiology, Erasmus Medical Center, Rotterdam, The Netherlands; 3 Department of Pediatrics, Erasmus Medical Center, Rotterdam, The Netherlands; 4 Weil Cornell Medical College – Qatar, Doha, Qatar; 5 Department of Biological Psychology, VU University, Amsterdam, The Netherlands; 6 Department of Obstetrics and Gynecology, Erasmus Medical Center, Rotterdam, The Netherlands; 7 Department of Public Health, Erasmus Medical Center, Rotterdam, The Netherlands; 8 Department of Biostatistics, Erasmus Medical Center, Rotterdam, The Netherlands; University of Hong Kong, Hong Kong

## Abstract

**Background:**

The objective was to estimate the heritability for height and weight during fetal life and early childhood in two independent studies, one including parent and singleton offsprings and one of mono- and dizygotic twins.

**Methods:**

This study was embedded in the Generation R Study (n = 3407, singletons) and the Netherlands Twin Register (n = 33694, twins). For the heritability estimates in Generation R, regression models as proposed by Galton were used. In the Twin Register we used genetic structural equation modelling. Parental height and weight were measured and fetal growth characteristics (femur length and estimated fetal weight) were measured by ultrasounds in 2^nd^ and 3^rd^ trimester (Generation R only). Height and weight were assessed at multiple time-points from birth to 36 months in both studies.

**Results:**

Heritability estimates for length increased from 2^nd^ to 3^rd^ trimester from 13% to 28%. At birth, heritability estimates for length in singletons and twins were both 26% and 27%, respectively, and at 36 months, the estimates for height were 63% and 72%, respectively. Heritability estimates for fetal weight increased from 2^nd^ to 3^rd^ trimester from 17% to 27%. For birth weight, heritability estimates were 26% in singletons and 29% in twins. At 36 months, the estimate for twins was 71% and higher than for singletons (42%).

**Conclusions:**

Heritability estimates for height and weight increase from second trimester to infancy. This increase in heritability is observed in singletons and twins. Longer follow-up studies are needed to examine how the heritability develops in later childhood and puberty.

## Introduction

Heritability is the proportion of variability of a phenotype that can be explained by polymorphic genes. Total adult body height is a highly heritable trait, with an estimated heritability of about 80 to 90% [1]. The heritability of weight and body mass index is considered to be generally lower, but estimates can still be as high as 85% [2]. Heritability can be estimated through twin studies, where resemblance of monozygotic (MZ) and dizygotic (DZ) twin pairs is compared. A first impression of heritability can be obtained by doubling the difference between the MZ and DZ correlations [1], [3], [4], [5].

It has been questioned whether twin studies are suitable for estimating heritability of early growth, since early growth patterns in twins are quite different from singleton growth patterns [6]. Galton suggested that the heritability of height can also be estimated by regressing the height of offspring against the mid-parental height [7]. Regression on mid-parental values has the additional advantage that the estimated heritability is not affected by assortative mating [4]. Cole demonstrated that the accuracy of this method could be improved by using standard deviation scores (SDS) instead of height measurements in centimetres [8], [9]_ENREF_9. Heritability estimates on anthropometrics are often obtained from single cross-sectional measurements [1], [10], [11], [12], [13], [14]. Few studies focused on anthropometrics throughout early, especially fetal, life [15], [16], [17], [18], [19]. As compared to final adult height and weight, the heritability of size-at-birth is low [17], [18], [20], [21]. In a parent-offspring cohort among over a 100000 families, the fetal genetic contribution to birth weight was suggested to be 31%, while for birth length it was 27% [20]. Also, a previous study demonstrated that heritability of birth weight decreased between 25 and 42 weeks of gestation from 52% to 30% [17]. These findings suggest that the genetic contribution to growth should decrease from second trimester until birth, when maternal-uterine factors are more dominant, and then increase from birth to adulthood. Furthermore, Ounsted et al. showed that when uterine constraint is relaxed the Mendelian laws of inheritance are followed [22]. However, as uterine constraint increases there is evidence for transmission of constraint through the female line [22].

We hypothesized that the heritability of weight and height would be relatively high during the first half of pregnancy, lower during third trimester or at birth and gradually increase throughout early childhood. We tested this hypothesis in the Generation R Study, a population-based prospective study from early fetal life onwards among 3407 Caucasian singletons and their parents, and the Netherlands Twin Register, a large twin cohort of the same population of 33694 individuals.

## Methods

### Study designs and cohorts

This study was embedded in the Generation R Study and the Netherlands Twin Register. Generation R is a population-based prospective cohort study from early fetal life onwards based in Rotterdam, the Netherlands, designed to identify early determinants of growth, development and health from fetal life until young adulthood. The Twin Register was established around 1987 to examine the genetic and environmental contribution to health and disease. Newborn twins are registered at birth by their parents. Both studies have been described previously in detail [23], [24], [25], [26]. These studies have been approved by the Medical Ethics Committees of the Erasmus Medical Center, Rotterdam and the Vrije Universiteit, Amsterdam. Written informed consent was obtained from all participants and/or their parent(s)/guardian(s).

### Population for analysis

#### The Generation R Study

Analyses were restricted to parent-child trio's of singleton pregnancies from Dutch or other European Caucasian ethnicity with parental height and weight data available (n = 3407). Due to miscarriage and prenatal loss to follow-up, data at birth were collected for 3370 newborns. The prenatal follow-up rate was 95%. For the postnatal analyses, 9% of the study population lived outside the study area, leaving 3084 subjects. The postnatal overall follow-up rate was 73%.

#### The Netherlands Twin Register

Analyses were performed in 33694 twins (from 16848 twin pairs) with a least one measurement of weight or height. There were 4956 MZMale, 5626 DZMale, 5562 MZFemale, 5148 DZFemale, and 6328 DZ MF and 6076 DZ FM. Zygosity status was based on blood group/DNA group polymorphisms for 1563 same-sex pairs. For the remaining same-sex twin pairs zygosity was assessed using items about physical similarity and frequency of confusion of the twins by family and strangers [27], collected in surveys at 3, 5, 7, 10, and 12 years. Ethnicity was derived from country of birth parents. For all included twins both parents were born in the Netherlands (96.5%) or other Western countries (3.5%). Weight and height were obtained from surveys mailed to the parents when the twins were 1, 2, and 3 years, with response rates of 83%, 80%, and 68% respectively.

### Growth measurements in Generation R

#### Fetal growth measurements

Fetal ultrasound examinations were carried out at the research centers in each trimester of pregnancy [28], [29]. These fetal ultrasound examinations were used for both establishing gestational age and assessing fetal growth characteristics. Crown-rump length was used for pregnancy dating in early pregnancy (gestational age until 12 weeks and 5 days, crown-rump length smaller than 65 mm) and biparietal diameter was used for pregnancy dating thereafter (gestational age from 12 weeks and 5 days onwards, biparietal diameter larger than 20 mm). For the present study, we measured fetal head circumference (HC), abdominal circumference (AC) and femur length (FL) to the nearest millimeter using standardized ultrasound procedures in second and third trimester (median ages: 20.5 weeks (90% range: 19.0–22.6) and 30.4 weeks (90% range: 28.9–32.4), respectively) [29]. Estimated fetal weight (EFW) was calculated using the formula by Hadlock (log_10_ EFW = 1.5662−0.0108 (HC)+0.0468 (AC)+0.171 (FL)+0.00034 (HC)^2^−0.003685 (AC * FL)) [30]. Ultrasound examinations were performed using an Aloka® model SSD-1700 (Tokyo, Japan) or the ATL-Philips® Model HDI 5000 (Seattle, WA, USA). Fetal measurements in early pregnancy were not included as growth characteristics because these ultrasound examinations were primarily performed to establish gestational age.

#### Parental and childhood growth measurements

In Generation R, maternal pre-pregnancy weight was obtained through questionnaire at the enrolment in the study. In addition, weight (during pregnancy) was assessed at the research centre. Correlation of pre-pregnancy weight obtained by questionnaire and weight measured at enrolment (median gestational age 13.5 (90% range 10.8–21.4) was 0.97 (P<0.001). Maternal height and paternal weight and height were also measured at the research center using standardized procedures.

Date of birth, birth anthropometrics (length and weight) and offspring sex were obtained from community midwife and hospital registries. Well-trained staff in community health centers obtained postnatal growth characteristics (length and weight) using standardized procedures [23]. Based on the routine health care program (Youth Health Service), visits for these growth characteristics were grouped into eight age periods. Median (90% range) ages (in months) of these periods were: 1.1 (0.9–1.6); 2.2 (2.0–2.9); 3.3 (3.1–3.9); 4.4 (4.0–4.9); 6.1 (5.4–7.3); 14.2 (13.6–15.9); 24.7 (23.4–27.3); and 36.5 (36.5–39.5).

### Growth measurements in the Twin Register

No fetal growth were available in the Twin Register. Parents of twins reported on sex, gestational age, birth weight and birth length in survey-1, which is collected after parents register their twins (age <1 years). In survey-2 and 3, mailed out when the twins were respectively 2 and 3 years old, a parent was asked to report height and weight as measured by the routine health care program in the Netherlands (Youth Health Services; up until the age of approximately 3.5 years). Based on this program, visits for these growth measurements were grouped similarly to those of Generation R [31], [32]. Median (90% range) ages (in months) of these periods were: 1.2 (0.9–1.6); 2.3 (1.9–2.9); 3.3 (3.0–3.9); 4.4 (4.0–4.9); 6.1 (5.5–7.0); 14.6 (13.8–15.6); 24.6 (23.5–26.7); and 36.7 (35.4–39.0).

### Statistical analysis

The creation of Standard Deviation Scores (SDS) for fetal growth in Generation R is described in the File S1. In both Generation R and the Twin Register, weight and height after birth were converted to SDS using the software package growth analyzer 3.5 containing the Dutch reference growth charts for the general population from 1997 for all postnatal growth measures [31], [32]. In Generation R, mid-parental height and weight standard deviation scores (SDS) were created by taking the average of the two parents. For maternal weight, pre-pregnancy weight was used. Subsequently, the heritability estimate (h^2^) was determined using the method of Galton [7]. The slope of the regression line (β1) approximates the heritability when the height or weight of the offspring is regressed against the average height or weight in the parents. Since fetal body length cannot be measured, femur length in second and third trimester was used as a proxy for body length prenatally [33]. Finally, to distinguish the between the paternal and maternal contributions, we regressed the height of the child (in SDS) against both paternal and maternal height separately (in SDS) using a single parent-offspring model. Statistical analyses were performed using the Statistical Package of Social Sciences version 17.0 for Windows (SPSS Inc, Chicago, IL, USA) and R version 2.10.1 (The R Foundation for Statistical Computing).

The genetic analyses of the twin data were carried out using genetic structural equation modeling in Mx, using maximum likelihood estimation [34]. By using data from twin pairs, it is possible to divide the total variation of weight and height into variance due to additive genetic factors (A), shared environmental factors (C), and due to environmental factors not shared by twins (E). If the MZ resemblance is twice as large as the DZ resemblance, the trait is influenced by additive genetic factors, because the only difference between the two zygosity groups is in genetic relatedness. If the DZ resemblance is the same or larger than half the MZ resemblance, then a trait is influenced by shared environmental factors. Shared environmental factors include the in utero experiences and postnatal shared experiences. An assumption of the twin method is that MZ and DZ twin pairs share these experiences to the same extent. Differences between MZ twins are attributable to their non-shared experiences including measurement error. For all growth measures a full model with A, C, and E factors was applied and the 95% confidence intervals were estimated. The A, C and E influences were specified as latent factors in the structural model and these latent factors influence observed data on height and weight in MZ and DZ twins. The A factors are correlated 1 in MZ and 0.5 in DZ twin pairs; the C factors correlate perfectly by definition in both pairs of twins. To estimate the heritability, the variance explained the A factors is divided by the total phenotypic variance. We have previously demonstrated that maternal smoking does not change the heritability of birth weight [21]. Finally, in both Generation R and the Twin Register, gestational age was modelled as a fixed effect, because a part of the variation in birth weight and length may be explained by the differences in gestational age.

## Results

The parental anthropometric characteristics in Generation R are shown in Table 1 and Table 2 gives the fetal (Generation R) and childhood (both studies) growth characteristics. The twins from the Twin Register were shorter and lighter throughout early childhood than the singletons from Generation R, starting with a lower birth weight and length and a shorter gestational age at birth.

**Table 1:** Parental characteristics in the Generation R Study.

Parental characteristics [5]	N	Mean (SD)/Median (90% range)
**Maternal characteristics**		
Age (years)	3407	31.5 (4.1)
Height (cm)	3405	170.6 (6.4)
Standard deviation score	3405	0.00 (1.00)
Pre-pregnancy weight (kg)	3394	68.0 (55.0, 94.0)
Standard deviation score	3394	−0.09 (−1.24, 1.90)
**Paternal characteristic**		
Age (years)	3406	33.6 (4.9)
Height (cm)	3407	184.3 (7.0)
Standard deviation score	3407	0.00 (1.00)
Weight (kg)	3401	84.5 (67.0, 108.0)
Standard deviation score	3401	−0.08 (−1.46, 1.79)
**Mid-parental characteristics**		
Height (cm)	3404	177.4 (5.2)
Standard deviation score	3404	0.00 (0.78)
Weight (kg)	3388	77.0 (64.0, 95.5)
Standard deviation score	3388	−0.08 (−1.11, 1.40)

*Values represent means (standard deviation) or median (90% range).*

**Table 2:** Child characteristics in the Generation R Study and the Netherlands Twin Register.

	The Generation R Study		Netherlands Twin Register	
Child characteristic	N	Mean (SD)		
**Sex (% boys)**	3370	50.3%	N/A	N/A
**Second trimester**				
Gestational age (weeks)	3171	20.5 (19.0–22.6)	N/A	N/A
Femur length (mm)	3171	33.3 (3.3)	N/A	N/A
Estimated fetal weight (grams)	3154	380 (87)	N/A	N/A
**Third trimester**				
Gestational age (weeks)	3248	30.4 (28.9–32.4)	N/A	N/A
Femur length (mm)	3203	57.5 (3.0)	N/A	N/A
Estimated fetal weight (grams)	3234	1638 (260)	N/A	N/A
**Birth**				
Gestational age (weeks)	3267	40.1 (36.7–42.1)	33528	37.0 (32.0–40.0)
Length (cm)	2282	50.5 (2.3)	24450	46.7 (3.6)
Weight (grams)	3253	3514 (509)	33102	2503 (546)
**1 month**				
Age (months)	2409	1.1 (0.9–1.6)	23644	1.2 (0.9–1.6)
Length (cm)	2026	54.5 (2.4)	13647	51.7 (2.5)
Weight (grams)	2407	4454 (619)	23481	3596 (558)
**2 months**				
Age (months)	2084	2.2 (2.0–2.9)	26732	2.3 (1.9–2.9)
Length (cm)	1501	58.6 (2.7)	17990	55.2 (3.0)
Weight (grams)	2081	5540 (748)	26565	4553 (728)
**3 months**				
Age (months)	2112	3.3 (3.1–3.9)	26735	3.3 (3.0–3.9)
Length (cm)	1777	61.6 (2.5)	24639	58.6 (2.9)
Weight (grams)	2109	6271 (763)	26592	5426 (789)
**4 months**				
Age (months)	1897	4.4 (4.0–4.9)	26399	4.4 (4.0–4.9)
Length (cm)	1398	64.2 (2.5)	22859	61.5 (2.9)
Weight (grams)	1892	6906 (788)	26298	6158 (828)
**6 months**				
Age (months)	2698	6.1 (5.4–7.3)	30042	6.1 (5.5–7.0)
Length (cm)	2394	67.7 (2.6)	28341	65.5 (2.9)
Weight (grams)	2686	7814 (857)	29922	7133 (900)
**14 months**				
Age (months)	2520	14.2 (13.6–15.9)	25994	14.6 (13.8–15.6)
Height (cm)	2503	78.3 (2.7)	25370	77.7 (3.0)
Weight (grams)	2502	10527 (1089)	25780	10150 (1149)
**24 months**				
Age (months)	2276	24.7 (23.4–27.3)	16157	24.6 (23.5–26.7)
Height (cm)	2240	88.3 (3.4)	15771	87.8 (3.6)
Weight (grams)	2272	12924 (1422)	15987	12489 (1458)
**36 months**				
Age (months)	2063	36.5 (35.3–39.5)	17305	36.7 (35.4–39.0)
Height (cm)	2029	97.5 (3.8)	17049	97.2 (4.0)
Weight (grams)	2040	15223 (1728)	16987	14757 (1765)

*Values represent means (standard deviation) or percentages.*

**Figure 1:**
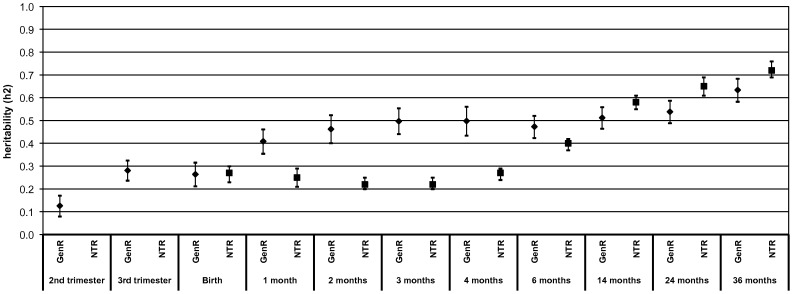
Heritability of height from second trimester of pregnancy until the age of 36 months. Values reflect heritability estimates (95% confidence interval). Model in the Generation R Study: height (SDS) = β0+β1 * mid-parental height (SDS). Here the slope ‘β1’ is equal to the heritability ‘h^2^’ [7]. Prenatally height is femur length (SDS). Postnatal growth is additionally adjusted for gestational age at birth. Model in the Netherlands Twin Register: Full twin model with additive genetic (A), shared environmental (C) and non-shared environmental (E) factors.

**Figure 2:**
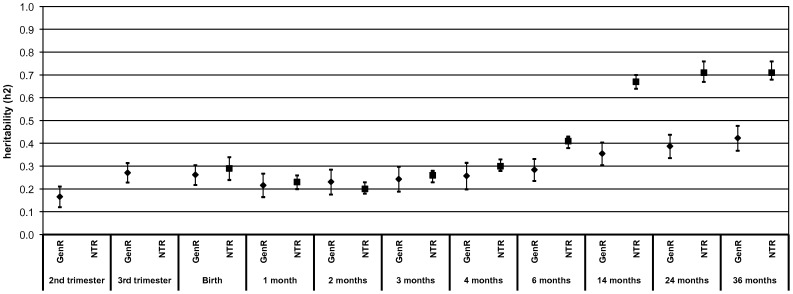
Heritability of weight from second trimester of pregnancy until the age of 36 months. Values reflect heritability estimates (95% confidence interval). Model in the Generation R Study: weight (SDS) = β0+β1 * mid-parental weight (SDS). Here the slope ‘β1’ is equal to the heritability ‘h^2^’ [7]. Prenatally weight is estimated fetal weight (SDS). Postnatal growth is additionally adjusted for gestational age at birth. Model in the Netherlands Twin Register: Full twin model with additive genetic (A), shared environmental (C) and non-shared environmental (E) factors.

Figures 1 and 2 show the heritability estimates from second trimester to 36 months for height and weight, respectively, in the Generation R Study and the Netherlands Twin Register. Also, Table 3 gives the estimates for additive genetic (A), shared environmental (C) and non-shared environmental (E) factors from the Twin Register. The heritability estimates for birth weight and height are comparable in both studies. In the Generation R Study, the heritability for height increased strongly from second to third trimester (13% (95% confidence interval (CI): 8%, 17%) to 28% (95% CI: 24%, 32%). In the first month of life, the heritability of height increased rapidly from 26% at birth (95% CI: 21%, 32%) to 41% (95% CI: 36%, 46%) at 1 month. This increase was followed by a more gradual increase to a heritability of 63% (95% CI: 58%, 68%) at 36 months. In the twins, however, no strong increase in heritability estimates for height was observed in the first months of postnatal life, as was seen in Generation R. The main increase occurred between 6 and 14 months, eventually leading to a comparable heritability estimate to that in Generation R at the age of 36 months (72% (95% CI: 69%, 76%).

**Table 3:** Heritability of height and weight from birth until the age of 36 months in the Netherlands Twin Register.

	Height			Weight		
	A	C	E	A	C	E
Birth	27 (23–30)	46 (43–49)	27 (26–29)	29 (24–34)	22 (18–25)	49 (48–51)
1 month	25 (21–29)	52 (48–55)	23 (22–25)	23 (20–26)	55 (52–57)	22 (21–24)
2 months	22 (20–25)	59 (56–61)	19 (18–20)	20 (18–23)	61 (59–63)	19 (18–20)
3 months	22 (20–25)	59 (57–61)	19 (18–19)	26 (23–28)	55 (53–58)	19 (18–20)
4 months	27 (24–29)	56 (54–58)	17 (16–18)	30 (28–33)	50 (48–52)	20 (19–21)
6 months	40 (37–42)	45 (43–47)	15 (15–16)	41 (38–43)	40 (38–42)	19 (18–20)
14 months	58 (55–61)	28 (25–31)	14 (13–15)	67 (64–70)	17 (15–21)	16 (15–16)
24 months	65 (61–69)	23 (20–27)	12 (11–13)	71 (67–76)	13 (9–17)	16 (15–17)
36 months	72 (69–76)	17 (14–21)	11 (10–11)	71 (68–76)	14 (10–17)	15 (14–16)

*Values reflect heritability estimates (95% confidence interval).*

*Model: Full twin model with additive genetic (A), shared environmental (C) and non-shared environmental (E) factors.*

Regarding weight, the heritability had a similar pattern as fetal height increasing strongly from second to third trimester (from 17% (95% CI: 12%, 21%) to 27% (95% CI: 23%, 31%)) in Generation R. After birth, the heritability for weight showed a slight decline followed by a gradual increase to a heritability of 42% (95% CI: 37%, 48%) at 36 months. For the twins, the heritability estimates up to the age of 4 months were similar to those in Generation R, showing a marginal decline in the first 3 months. However, in the twins the heritability estimates were markedly higher after the age of six months, resulting in a heritability estimate of 71% (95% CI: 68, 76%) at the age of 36 months.

The parental effects of each parent separately as measured in the Generation R Study are shown in Table 4. For second and third trimester estimated fetal weight, the heritability was higher for maternal weight than paternal weight (second trimester: 13% (95% CI: 10%, 17%) for maternal weight versus 7% (95% CI: 4%, 11%) for paternal weight; and third trimester: 22% (95% CI: 18%, 25%) for maternal weight versus 12% (95% CI: 8%, 15%) for paternal weight). The maternal effects for weight decreased after birth (22% (95% CI: 18%, 25%) at birth to 14% (95% CI: 10%, 18%) at 1 month), after which there were no large differences in weight estimates between the parents until the age of 3 years. For height, the maternal effects were consistently higher as compared to those based on paternal height from second trimester to 3 years of age.

**Table 4:** Estimates for height and weight from second trimester to 36 months stratified by parent.

Age	Height		Weight	
	Mother	Father	Mother	Father
Second trimester	0.097 (0.062, 0.132)	0.053 (0.028, 0.087)	0.131 (0.096, 0.166)	0.070 (0.035, 0.105)
Third trimester	0.196 (0.161, 0.230)	0.140 (0.106, 0.174)	0.215 (0.181, 0.248)	0.116 (0.082, 0.150)
Birth	0.178 (0.137, 0.219)	0.139 (0.098, 0.179)	0.218 (0.184, 0.252)	0.104 (0.069, 0.138)
1 month	0.275 (0.233, 0.316)	0.217 (0.175, 0.259)	0.140 (0.099, 0.181)	0.126 (0.086, 0.165)
2 months	0.331 (0.278, 0.379)	0.220 (0.170, 0.269)	0.142 (0.098, 0.186)	0.144 (0.101, 0.187)
3 months	0.342 (0.298, 0.387)	0.244 (0.199, 0.289)	0.131 (0.088, 0.174)	0.166 (0.123, 0.208)
4 months	0.324 (0.274, 0.375)	0.265 (0.215, 0.315)	0.138 (0.082, 0.185)	0.177 (0.132, 0.222)
6 months	0.324 (0.287, 0.363)	0.237 (0.198, 0.276)	0.185 (0.143, 0.220)	0.170 (0.133, 0.208)
14 months	0.315 (0.278, 0.353)	0.287 (0.249, 0.325)	0.215 (0.174, 0.255)	0.218 (0.180, 0.257)
24 months	0.338 (0.299, 0.377)	0.308 (0.268, 0.347)	0.243 (0.202, 0.284)	0.228 (0.188, 0.269)
36 months	0.392 (0.351, 0.433)	0.352 (0.312, 0.393)	0.273 (0.230, 0.316)	0.240 (0.197, 0.283)

Model for mothers: height or weight (SDS) = β0+β1 * maternal height or weight (SDS).

Model for fathers: height or weight (SDS) = β0+β1 * paternal height or weight (SDS).

*The estimates are based on β1 for single parent-offspring estimates.*

*Heritability estimates are given with 95% confidence interval.*

*Prenatally height is femur length (SDS) and weight is estimated fetal weight (SDS).*

## Discussion

In this study we estimated the heritability of body size from second trimester until the postnatal age of 36 months using data from two different studies among parental-child trios and twins. In singletons, the heritability estimates for height and weight increased from 2^nd^ trimester to 36 months from 13% to 63% and from 20% to 42%, respectively. A similar increase between birth and 36 months (27% to 72%) was found for height when analyzing data from MZ and DZ twins. For both birth length (26% in singletons and 27% in twins) and birth weight (26% in singletons and 29% in twins) the heritability estimates were remarkably similar in both studies. The only difference in heritability estimates between the twin and singleton sample is seen for weight at 36 months. Heritability estimates were higher (71%) in twins than in singletons (42%).

Prior to the study, we had hypothesized that the heritability of height and weight would be relatively high in the first half of pregnancy, lower in third trimester or birth and gradually increase throughout early childhood. However, we observed that the heritability estimates for height and weight were considerably lower in second trimester than third trimester. Previously, the heritability of weight at 25 weeks of gestation was estimated to be 52%, which is much higher than the 17% found in the current study [17]. Genes may indeed not play such an important role in early fetal growth. On the other hand, at mid-pregnancy random measurement error may have led to an underestimation of the heritability estimates.

For height the heritability estimates at birth was around 26–27% is both studies. In Generation R, the heritability estimates increased strongly during the first month postnatally. Studies have shown that children tend to catch-up or catch-down in the first years after birth, after which growth generally continues along the same percentile until the individual reaches the target height in adulthood [35]. Changes in early postnatal growth rates are influenced by a drive to compensate for prenatal fetal growth restriction or growth enhancement caused by the maternal-uterine environment [35]. These estimates would indicate that a significant proportion of this catch-up or catch-down growth in height would occur already in the first few weeks of postnatal life. Nonetheless, for both weight and height, heritability estimates at the age of 36 months were lower than those estimated in other samples in adulthood [1], [2]. We expect that these values would increase further into adulthood. This increase in heritability might be expected to occur before or during puberty, since the onset of the pubertal growth spurt and the age of peak height velocity have been shown to be highly heritable (91% and 93%, respectively) [14].

Regarding weight, a previous study demonstrated that heritability of birth weight decreased between 25 and 42 weeks of gestation from 52% to 30% [17]. In both the Generation R and the Twin Register, the heritability of birth weight was similar (26% and 30%, respectively). Using directly measured fetal growth characteristics, we showed that the heritability did not increase in the last ten weeks of pregnancy and was almost constant throughout the first 4 months in postnatal life. This lack of increase could be explained by a more dominant role for the uterine environment on weight gain in third trimester fetal growth and that this effect continues into early postnatal life. This idea is supported by the fact that the influence of the shared environment (C) in the Twin Register stayed fairly constant between 1 and 4 month (50–61%) and then dropped sharply to 17% at the age of 14 months. Though both studies showed clear increases of heritability estimates for weight during early childhood, at the age of 36 months the heritability was estimated to be 42% and 71% in Generation R and the Twin Register respectively.

Some methodological issues need to be considered. In our study the postnatal follow-up rate was 73%. It is unlikely, however, that this loss to follow-up biased our results, since one can assume that the follow-up is independent of the heritability. To our knowledge, this is the first study that assessed the heritability of body size from fetal life onwards at several ages. In Generation R, the regression method from Galton [7], modified by Cole [9], allowed us to assess heritability of growth using parent-offspring trios. However, this method might lead to an overestimation of the genetic contribution in early growth. For example, we observed a relatively high heritability estimate for fetal weight and birth weight based on maternal weight as compared to paternal weight. Also, the heritability for postnatal length was consistently higher estimated for maternal height than for paternal height. A possible explanation is that mothers have a larger shared fetal and early postnatal environment with their offspring than fathers in early life. However, this does not explain why we did not observe a difference between the two parents regarding the heritability of weight. Maternal pre-pregnancy weight is known to be highly positively associated with fetal growth [36]. The relatively higher fetal weight heritability estimates for maternal weight than for paternal weight could be reflection of a shared maternal-fetal environment rather than shared genetic factors. Also, inheritance follows the Mendelian laws when there is little uterine constraint, but as uterine constraint increases there is evidence for transmission of constraint through the female line [22]. Another explanation is that not all the fathers in our study are the biological father, which would of course lead to a lower heritability estimate due to the decrease in shared genes. Finally, a parent-of-origin effect on early growth of genes regulating growth, as is known in the case of the genomic imprinting of the *IGF2* gene might explain part of the difference in heritability estimates between the parents [20], [37].

Estimating heritability by either Galton's method that makes use of mid-parent – offspring data or the classical twin method that compares identical and fraternal twins both have their own strengths and shortcomings. Galton's method makes the strong assumption that the same genes affect parents' and offspring's phenotype [4]. Growth genes are known to affect anthropometrics differently in various stages of development [38], which could thus lead to a downward bias of the heritability estimate. Regarding the twin method, it remains unknown whether twin studies are suitable for estimating heritability of early growth, since early growth patterns in twins are quite different from singleton growth patterns [6]. Also, though rare, this method can be prone bias due to a high discrepancy of birth weight in MZ pairs. Thus, the increasing trend in heritability in the current study can also be due to the increasing genetic correlation with adult height (Galton's method) and/or the decreasing environmental variation related to twin pregnancies (twin method). One could hypothesize that a different study design could reduce these types of potential biases, for example by comparing third generation data from MZ and DZ twins.

In conclusion, the current study demonstrated that the heritability of height and weight increases from second trimester to infancy in two independent studies from the same population, using two different methods to estimate heritability. Longer follow-up studies are necessary to examine how the heritability develops in later childhood and puberty.

## Supporting Information

File S1 Creation of standard deviation scores in the Generation R Study.
(DOCX)

## References

[1] Silventoinen K, Sammalisto S, Perola M, Boomsma DI, Cornes BK (2003). Heritability of adult body height: a comparative study of twin cohorts in eight countries.. Twin Res.

[2] Yang W, Kelly T, He J (2007). Genetic epidemiology of obesity.. Epidemiol Rev.

[3] Schousboe K, Willemsen G, Kyvik KO, Mortensen J, Boomsma DI (2003). Sex differences in heritability of BMI: a comparative study of results from twin studies in eight countries.. Twin Res.

[4] Falconer DS, Mackay TFC (1996). Introduction to quantitative genetics.

[5] Boomsma D, Busjahn A, Peltonen L (2002). Classical twin studies and beyond.. Nat Rev Genet.

[6] Phillips DI (1993). Twin studies in medical research: can they tell us whether diseases are genetically determined?. Lancet.

[7] Galton F (1886). Regression towards mediocrity in hereditary stature.. Journal of the anthropological institute.

[8] Aulchenko YS, Struchalin MV, Belonogova NM, Axenovich TI, Weedon MN (2009). Predicting human height by Victorian and genomic methods.. Eur J Hum Genet.

[9] Cole TJ (2000). Galton's midparent height revisited.. Ann Hum Biol.

[10] Estourgie-van Burk GF, Bartels M, van Beijsterveldt TC, Delemarre-van de Waal HA, Boomsma DI (2006). Body size in five-year-old twins: heritability and comparison to singleton standards.. Twin Res Hum Genet.

[11] Ordonana JR, Rebollo-Mesa I, Gonzalez-Javier F, Perez-Riquelme F, Martinez-Selva JM (2007). Heritability of body mass index: a comparison between the Netherlands and Spain.. Twin Res Hum Genet.

[12] Hur YM, Luciano M, Martin NG, Boomsma DI, Iacono WG (2005). A comparison of twin birthweight data from Australia, the Netherlands, the United States, Japan, and South Korea: are genetic and environmental variations in birthweight similar in Caucasians and East Asians?. Twin Res Hum Genet.

[13] Clausson B, Lichtenstein P, Cnattingius S (2000). Genetic influence on birthweight and gestational length determined by studies in offspring of twins.. Bjog.

[14] Silventoinen K, Haukka J, Dunkel L, Tynelius P, Rasmussen F (2008). Genetics of pubertal timing and its associations with relative weight in childhood and adult height: the Swedish Young Male Twins Study.. Pediatrics.

[15] van Dommelen P, de Gunst MC, van der Vaart AW, Boomsma DI (2004). Genetic study of the height and weight process during infancy.. Twin Res.

[16] Silventoinen K, Bartels M, Posthuma D, Estourgie-van Burk GF, Willemsen G (2007). Genetic regulation of growth in height and weight from 3 to 12 years of age: a longitudinal study of Dutch twin children.. Twin Res Hum Genet.

[17] Gielen M, Lindsey PJ, Derom C, Smeets HJ, Souren NY (2008). Modeling genetic and environmental factors to increase heritability and ease the identification of candidate genes for birth weight: a twin study.. Behav Genet.

[18] Beardsall K, Ong KK, Murphy N, Ahmed ML, Zhao JH (2009). Heritability of childhood weight gain from birth and risk markers for adult metabolic disease in prepubertal twins.. J Clin Endocrinol Metab.

[19] Silventoinen K, Pietilainen KH, Tynelius P, Sorensen TI, Kaprio J (2007). Genetic and environmental factors in relative weight from birth to age 18: the Swedish young male twins study.. Int J Obes (Lond).

[20] Lunde A, Melve KK, Gjessing HK, Skjaerven R, Irgens LM (2007). Genetic and environmental influences on birth weight, birth length, head circumference, and gestational age by use of population-based parent-offspring data.. Am J Epidemiol.

[21] van Baal CG, Boomsma DI (1998). Etiology of individual differences in birth weight of twins as a function of maternal smoking during pregnancy.. Twin Res.

[22] Ounsted M, Scott A, Ounsted C (2008). Transmission through the female line of a mechanism constraining human fetal growth.. Int J Epidemiol.

[23] Jaddoe VW, van Duijn CM, van der Heijden AJ, Mackenbach JP, Moll HA (2008). The Generation R Study: design and cohort update until the age of 4 years.. Eur J Epidemiol.

[24] Jaddoe VW, Bakker R, van Duijn CM, van der Heijden AJ, Lindemans J (2007). The Generation R Study Biobank: a resource for epidemiological studies in children and their parents.. Eur J Epidemiol.

[25] Boomsma DI, de Geus EJ, Vink JM, Stubbe JH, Distel MA (2006). Netherlands Twin Register: from twins to twin families.. Twin Res Hum Genet.

[26] Bartels M, van Beijsterveldt CE, Derks EM, Stroet TM, Polderman TJ (2007). Young Netherlands Twin Register (Y-NTR): a longitudinal multiple informant study of problem behavior.. Twin Res Hum Genet.

[27] Rietveld MJ, van Der Valk JC, Bongers IL, Stroet TM, Slagboom PE (2000). Zygosity diagnosis in young twins by parental report.. Twin Res.

[28] Jaddoe VW, Mackenbach JP, Moll HA, Steegers EA, Tiemeier H (2006). The Generation R Study: Design and cohort profile.. Eur J Epidemiol.

[29] Verburg BO, Steegers EA, De Ridder M, Snijders RJ, Smith E (2008). New charts for ultrasound dating of pregnancy and assessment of fetal growth: longitudinal data from a population-based cohort study.. Ultrasound Obstet Gynecol.

[30] Hadlock FP, Harrist RB, Carpenter RJ, Deter RL, Park SK (1984). Sonographic estimation of fetal weight. The value of femur length in addition to head and abdomen measurements.. Radiology.

[31] Fredriks AM, van Buuren S, Wit JM, Verloove-Vanhorick SP (2000). Body index measurements in 1996-7 compared with 1980.. Arch Dis Child.

[32] Fredriks AM, van Buuren S, Burgmeijer RJ, Meulmeester JF, Beuker RJ (2000). Continuing positive secular growth change in The Netherlands 1955–1997.. Pediatr Res.

[33] Hadlock FP, Deter RL, Roecker E, Harrist RB, Park SK (1984). Relation of fetal femur length to neonatal crown-heel length.. J Ultrasound Med.

[34] Neale MC, Boker SM, Xie G, Maes HH (1999).

[35] Ong KK, Preece MA, Emmett PM, Ahmed ML, Dunger DB (2002). Size at birth and early childhood growth in relation to maternal smoking, parity and infant breast-feeding: longitudinal birth cohort study and analysis.. Pediatr Res.

[36] Ay L, Kruithof CJ, Bakker R, Steegers EA, Witteman JC (2009). Maternal anthropometrics are associated with fetal size in different periods of pregnancy and at birth. The Generation R Study.. Bjog.

[37] Tabano S, Colapietro P, Cetin I, Grati FR, Zanutto S (2010). Epigenetic modulation of the IGF2/H19 imprinted domain in human embryonic and extra-embryonic compartments and its possible role in fetal growth restriction.. Epigenetics.

[38] Sovio U, Mook-Kanamori DO, Warrington NM, Lawrence R, Briollais L (2011). Association between common variation at the FTO locus and changes in body mass index from infancy to late childhood: the complex nature of genetic association through growth and development.. PLoS Genet.

