# Supplementary material for: Heritability Estimates of Body Size in Fetal Life and Early Childhood
Source: PLoS One. 2012 Jul 25;7(7):e39901. doi: 10.1371/journal.pone.0039901 (PMC3405108; doi:10.1371/journal.pone.0039901)
Supplement: File S1 — Creation of standard deviation scores in the Generation R Study. (DOCX) [file pone.0039901.s001.docx]

**Supporting Information S1**

In the Generation R, study-based standard deviation scores (SDS) for fetal growth characteristics were created. These scores enable adjustment for gestational age avoiding the inclusion of non-linear functions of gestational age in models. Our approach for developing reference models for constructing SDS was based on the LMS model of Cole and Green ([39](#_ENREF_39)), as implemented in the GAMLSS software of Rigby and Stasinopoulos ([40](#_ENREF_40)). The LMS model of Cole and Green assumes that, after a Box-Cox transformation, the growth characteristic has a normal distribution. The acronym LMS stands for lambda, mu, and sigma. Mean and standard devia­tion of the distribution are allowed to change with gestational age, possibly by smooth curves, described by polynomials of splines. The formula for creating standard deviation scores is:


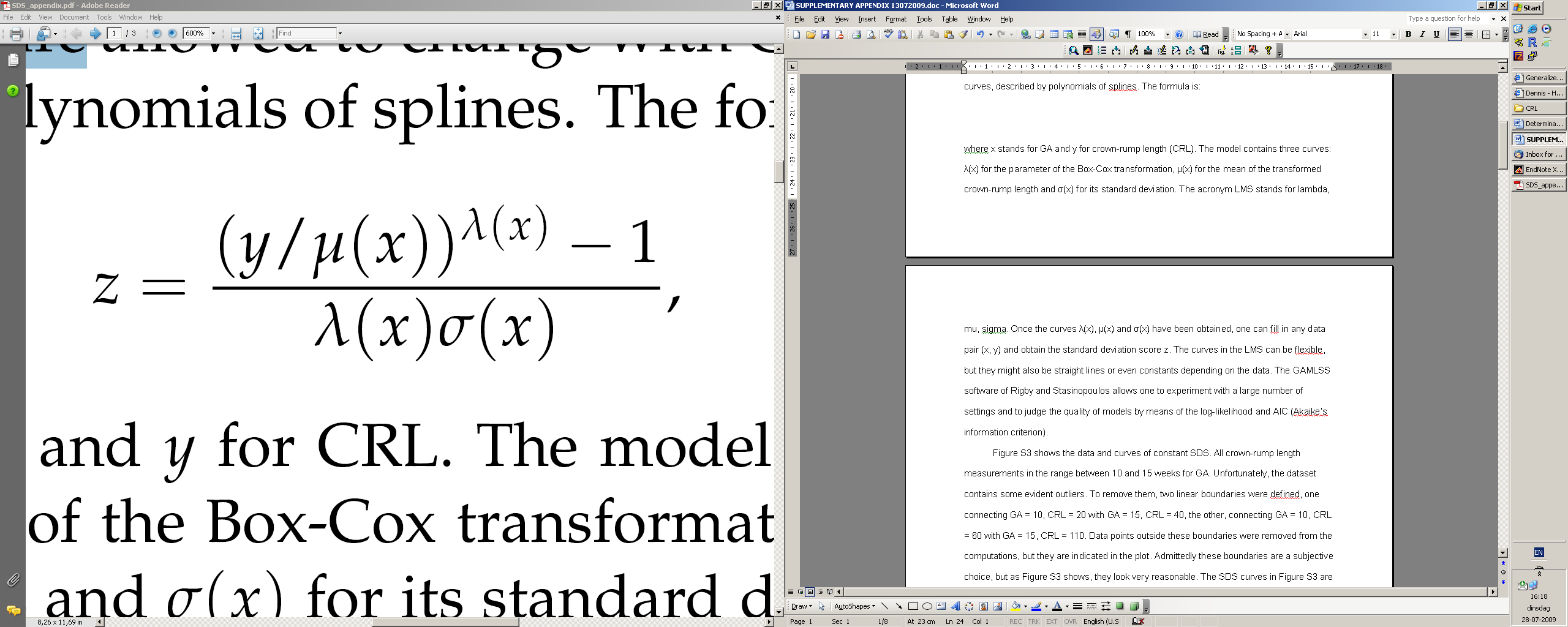


where x stands for (gestational) age and y for the growth parameter. The model comprises three curves: λ(x) for the parameter of the Box-Cox transformation, µ(x) for the mean of the transformed height or weight and σ(x) for its standard deviation. Once the curves λ(x), µ(x) and σ(x) have been obtained, one can ﬁll in any data pair (x, y) and obtain the standard deviation score z. The curves in the LMS can be ﬂexible, but they might also be straight lines or even constants depending on the data. The GAMLSS software of Rigby and Stasinopoulos allows one to experiment with a large number of settings and to judge the quality of models by means of the log-likelihood and Akaike’s information criterion (AIC).
